# Supplementary material for: The distinctive roles played by the superoxide dismutases of the extremophile Acinetobactersp. Ver3
Source: Sci Rep. 2022 Mar 12;12:4321. doi: 10.1038/s41598-022-08052-z (PMC8918354; doi:10.1038/s41598-022-08052-z)
Supplement: Supplementary file 1 — Supplementary Information 1. [file 41598_2022_8052_MOESM1_ESM.pdf]

## SUPPLEMENTARY MATERIAL

**Table S1. Prevalence of *sod* genes in the genus *Acinetobacter*.**

| Organism | No. strains | *sodB* | *sodA* | *sodC* | Genotype | % In database |
|---|---|---|---|---|---|---|
| *Acinetobacter albensis* | 1 | 1 | 0 | 1 | 1 | 83.4 |
| *Acinetobacter baumannii** | 163 | 1 | 0 | 1 | | |
| *Acinetobacter bohemicus* | 2 | 1 | 0 | 1 | | |
| *Acinetobacter bouvetii* | 2 | 1 | 0 | 1 | | |
| *Acinetobacter brisouii* | 2 | 1 | 0 | 1 | | |
| *Acinetobacter calcoaceticus* | 2 | 1 | 0 | 1 | | |
| *Acinetobacter cumulans* | 1 | 1 | 0 | 1 | | |
| *Acinetobacter defluvii* | 1 | 1 | 0 | 1 | | |
| *Acinetobacter gandensis* | 2 | 1 | 0 | 1 | | |
| *Acinetobacter genomosp.* | 3 | 1 | 0 | 1 | | |
| *Acinetobacter gerneri* | 1 | 1 | 0 | 1 | | |
| *Acinetobacter harbinensis* | 1 | 1 | 0 | 1 | | |
| *Acinetobacter indicus* | 7 | 1 | 0 | 1 | | |
| *Acinetobacter kookii* | 1 | 1 | 0 | 1 | | |
| *Acinetobacter kyonggiensis* | 1 | 1 | 0 | 1 | | |
| *Acinetobacter lactucae* | 1 | 1 | 0 | 1 | | |
| *Acinetobacter nosocomialis* | 14 | 1 | 0 | 1 | | |
| *Acinetobacter oleivorans* | 1 | 1 | 0 | 1 | | |
| *Acinetobacter parvus* | 3 | 1 | 0 | 1 | | |
| *Acinetobacter piscicola* | 1 | 1 | 0 | 1 | | |
| *Acinetobacter pitti* | 22 | 1 | 0 | 1 | | |
| *Acinetobacter pragensis* | 1 | 1 | 0 | 1 | | |
| *Acinetobacter radioresistens* | 4 | 1 | 0 | 1 | | |
| *Acinetobacter rudis* | 1 | 1 | 0 | 1 | | |
| *Acinetobacter schindleri* | 3 | 1 | 0 | 1 | | |
| *Acinetobacter seifertii* | 4 | 1 | 0 | 1 | | |

| | | | | | | |
|---|---|---|---|---|---|---|
| *Acinetobacter sp.* | 14 | 1 | 0 | 1 | | |
| *Acinetobacter tjernbergiae* | 1 | 1 | 0 | 1 | | |
| *Acinetobacter towneri* | 1 | 1 | 0 | 1 | | |
| *Acinetobacter venetianus* | 1 | 1 | 0 | 1 | | |
| *Acinetobacter beijerinckii* | 1 | 1 | 1 | 1 | 2 | 10.2 |
| *Acinetobacter bereziniae* | 1 | 1 | 2 | 1 | | |
| *Acinetobacter colistiniresistens* | 3 | 1 | 1 | 1 | | |
| *Acinetobacter dispersus* | 1 | 1 | 1 | 1 | | |
| *Acinetobacter equi* | 1 | 1 | 1 | 1 | | |
| *Acinetobacter guillouiae* | 1 | 1 | 1 | 1 | | |
| *Acinetobacter gyllenbergii* | 1 | 1 | 1 | 1 | | |
| *Acinetobacter idrijaensis* | 1 | 1 | 1 | 1 | | |
| *Acinetobacter johnsonii* | 5 | 1 | 1 | 1 | | |
| *Acinetobacter junii* | 3 | 1 | 1 | 1 | | |
| *Acinetobacter lwoffii* | 1 | 1 | 1 | 1 | | |
| *Acinetobacter marinus* | 1 | 1 | 1 | 1 | | |
| *Acinetobacter nectaris* | 1 | 1 | 1 | 1 | | |
| *Acinetobacter proteolyticus* | 1 | 1 | 1 | 1 | | |
| *Acinetobacter puyangensis* | 1 | 1 | 1 | 1 | | |
| *Acinetobacter sp.* | 3 | 1 | 1 | 1 | | |
| *Acinetobacter tandoii* | 1 | 1 | 1 | 1 | | |
| *Acinetobacter variabilis* | 3 | 1 | 1 | 1 | | |
| *Acinetobacter wuhouensis* | 2 | 1 | 1 | 1 | | |
| *Acinetobacter baylyi ADP1* | 1 | 1 | 2 | 0 | 3 | 6.1 |
| *Acinetobacter beijerinckii* | 1 | 1 | 1 | 0 | | |
| *Acinetobacter haemolyticus* | 7 | 1 | 2 | 0 | | |
| *Acinetobacter haemolyticus* | 4 | 1 | 1 | 0 | | |
| *Acinetobacter halotolerans* | 1 | 1 | 1 | 0 | | |
| *Acinetobacter qingfengensis* | 2 | 1 | 2 | 0 | | |

| | | | | | | |
|---|---|---|---|---|---|---|
| *Acinetobacter soli* | 1 | 1 | 2 | 0 | | |
| *Acinetobacter ursingii* | 1 | 1 | 2 | 0 | | |
| *Acinetobacter sp.* | 1 | 1 | 1 | 0 | | |
| *Acinetobacter apis* | 1 | 0 | 1 | 1 | N/A | 0.3 |

* *A. baumannii* strains 921, 3207 and 7835 encode a second *sodB* gene.

A comparative genomic analysis of *Acinetobacter* strains available at the NCBI database on September 16th, 2020 was performed. Only strains with genomic sequence assemblies classified as complete or scaffolds were included in the study. The genomic and proteomic data corresponding to 314 strains were extracted, and a local database was constructed. SODs encoded by *A. baumannii* ATCC 17978 were used as query to perform BLASTp-sequence similarity searches against the local database, using 40 % sequence identity and 90 % query coverage cut-off values in the case of Fe-MnSODs and 55 % sequence identity and 75 % query coverage for CuZnSODs. Signal P v5.0 (http://www.cbs.dtu.dk/services/SignalP/) was used to predict the presence or absence of signal peptide sequences in Fe-MnSODs and CuZnSODs.

**Table S2. Search of SODs encoded by *Acinetobacter spp*.** Local Blast analysis of SOD proteins in *Acinetobacter spp.*, using the FeSOD and CuZnSOD from *A. baumannii* ATCC17978 as query.

**Table S3. Strains and plasmids used in this work.**

| | **Description** | **Reference** |
|---|---|---|
| Strains | | |
| *Acinetobacter sp. Ver3* | | Ordoñez *et al.,* 2009 |
| *E. coli* | | |
| DH5a | Φ80 *lacZ*DM15 *rea*A1 *end*A1 *gyr*A96 *thi*-1 *hsd*R17 *sup*E44 *rel*A1 *deo*RD(*lac*ZYA-*arg*F) U169 F | Woodcock *et al.,* 1989 |
| QC774(DE3) | $F^-$ *ompT* ($r_B^-$ $m_B^-$) *gal dcm* λ(DE3) pLysS ($Cm^r$ ) | Carlioz & Touati 1986 |
| Plasmids | | |

| | | |
|---|---|---|
| pET32m | Expression vector, Amp$^R$, derived from pET32b, with a deletion on a enterokinase restriction site | Tabares *et al.,* 2006 |
| pET3228 | *oriR*(ColE1) *oriR*(f1) Amp$^R$ | Tabares *et al.,* 2006 |
| pESodB | pET3228 derivative expressing $^{AV3}$SodB | This work |
| pESodC$^{-p}$ | pET32m derivative expressing $^{AV3}$SodC without the sequence encoding the signal peptide | This work |
| pGEM®T-Easy | Cloning vector | Promega® |

**Table S4. Oligonucleotides used in this work.**

| **Primers** | **Sequence (5'–3')** |
|---|---|
| FMSOD3228F | 5'-CCATCCATGGCAACGATTACTTTACCAGCTCTTCC-3'[a] |
| FMSOD3228R | 5'-ACGGGAGCTCAATCTTATTTTTCTACGCCAGCTTCTTG-3'[b] |
| CSOD$^{sp}$32F | 5'-AGAAGGATCCGCAACGCAAAATACATCTGCATC-3'[c] |
| CSOD$^{sp}$32R | 5'-CCATCTCGAGGCTTAGCGTATAACACCACATGC-3'[d] |
| qFMSODF | 5'-AGGTATCTTCAACAACGCAGC-3' |
| qFMSODR | 5'-AGCAACTAACCAAGCCCAAC-3' |
| qCZSODF | 5'-ACCTGGATATCACGGGTTCC-3' |
| qCZSODR | 5'-CGCGTCAACATTTAACACTGG-3' |
| qrecAF | 5'-CTCAATATGCTCGCAAACTTGG-3' |
| qrecAR | 5'-GGTTAAGGCTGCTACAGAATCG-3' |
| qrpoBF | 5'-TGCAAACACGGTTCTTAGCC-3' |
| qrpoBR | 5'-CACCTGGACGCATTACCTTG-3' |

Underlined sequences correspond to recognition sites for restriction enzymes: [a]*Nco*I, [b]*Sac*I, [c]*Bam*HI, [d]*Xho*I, [e]*EcoR*I and [f]*Hind*III.

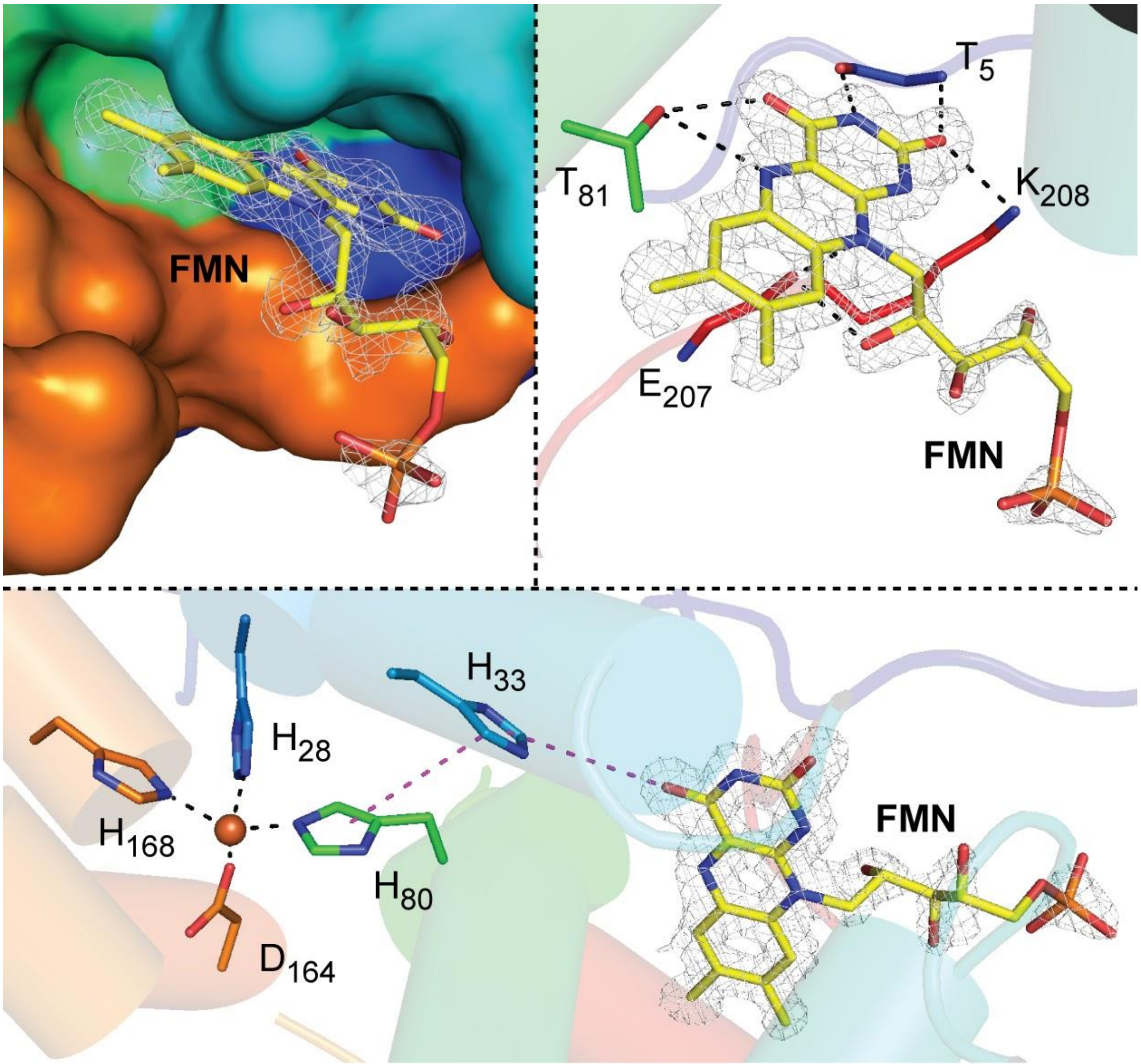


**Figure S1. A FMN binding site in $^{AV3}$SodB.** Upper panels: the binding pocket is shown in the protein in rainbow colours, in surface and ribbon representation. In the latter, the residues that establish polar interactions (black dashed lines) with the FMN are shown as sticks. The FMN molecule is also presented as sticks and the corresponding *2mFo–DFc* electron density (contoured to 1.0 σ) is displayed as a mesh. Bottom panel: relative position of the FMN molecule with respect to the active site of the enzyme. It is possible to estimate an electron transfer path (magenta dashed lines) (https://emap.bu.edu) between the FMN and residue His80 at the active site, which involves the His33. The iron ion is depicted as an orange sphere.

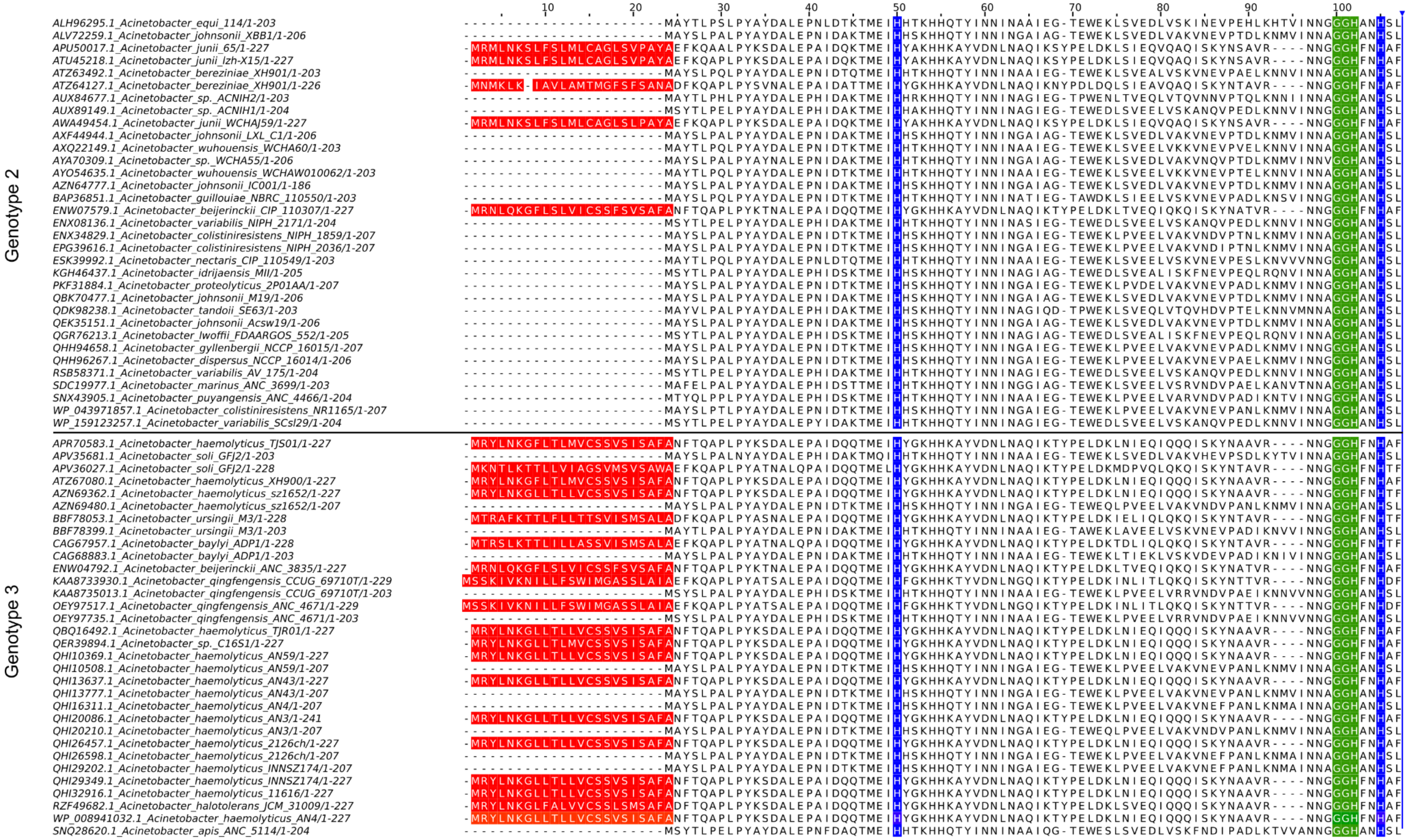

**Figure S2. Sequence alignment of MnSODs in *Acinetobacter spp.*.** In red, the putative signal peptides; in green, the characteristic MnSOD motif GGH; in blue, two first histidine residues from the metal binding site. Genotypes assigned in Table 2 are shown on the left. The C-terminal part of the proteins was intentionally deleted for a better visualization of the alignment.

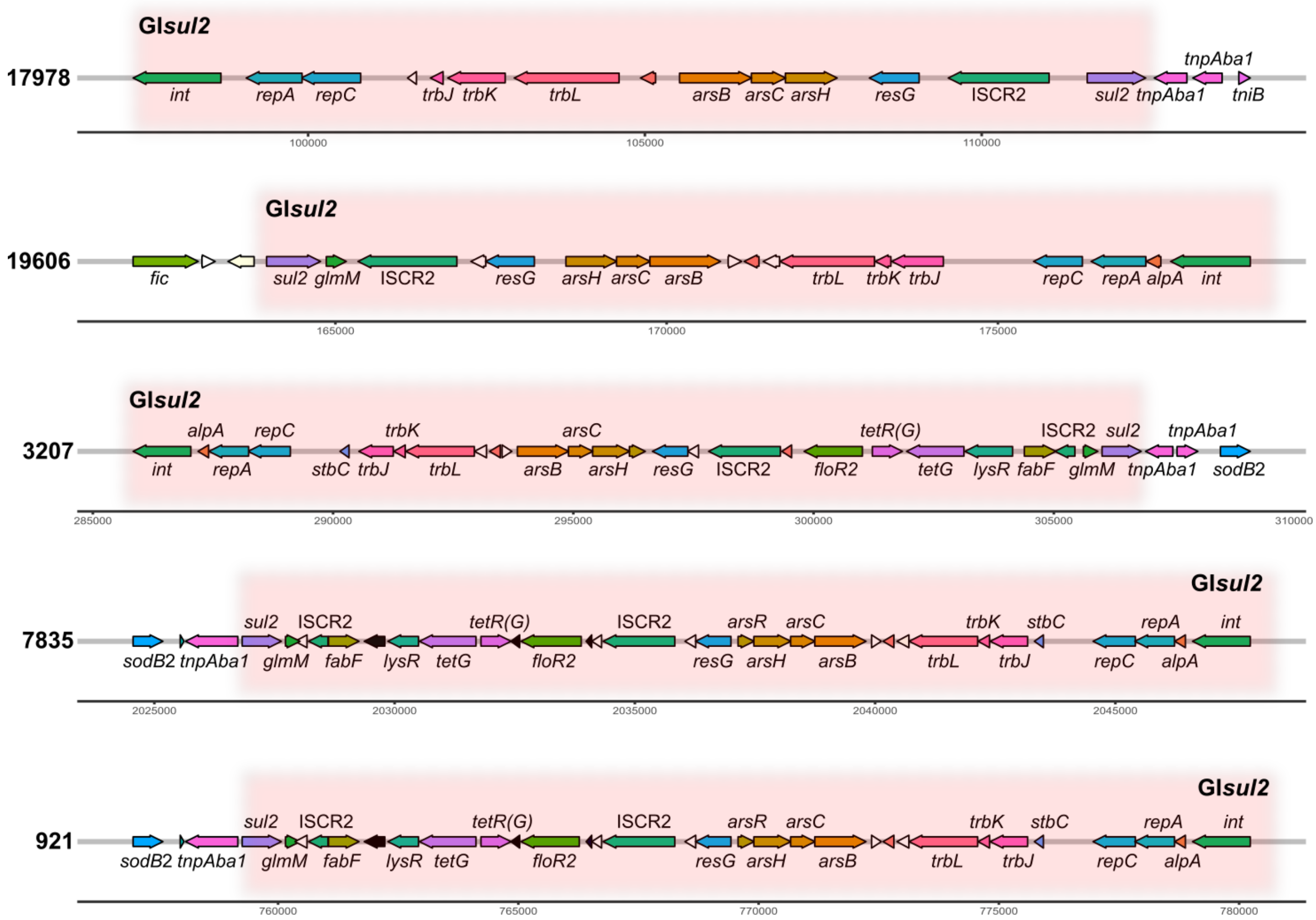


**Figure S3. Genomic localization of *sodB*2.** The *A. baumannii* strains 3207, 7835 and 921 encode a paralogous *sodB* gene (blue arrow), located close to a genomic island showing similarities to the GI*sul2* genomic island (pink box) found in *A. baumannii* ATCC17978 and ATCC19606 strains.

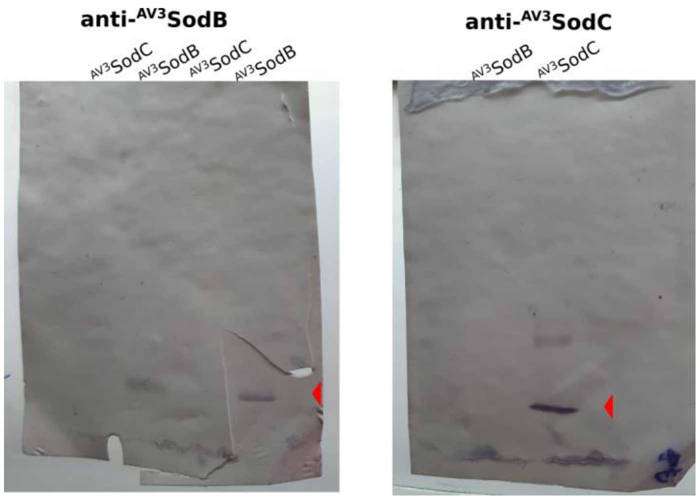

**Figure S4. Specificity test for anti-$^{AV3}$SodB and anti-$^{AV3}$SodC.** Each antibody was used against $^{AV3}$SodB and $^{AV3}$SodC in a western blot assay showing that there is no cross-reactivity. The anti-$^{AV3}$SodB test in the left blot was made in duplicate using the same amount of enzyme.

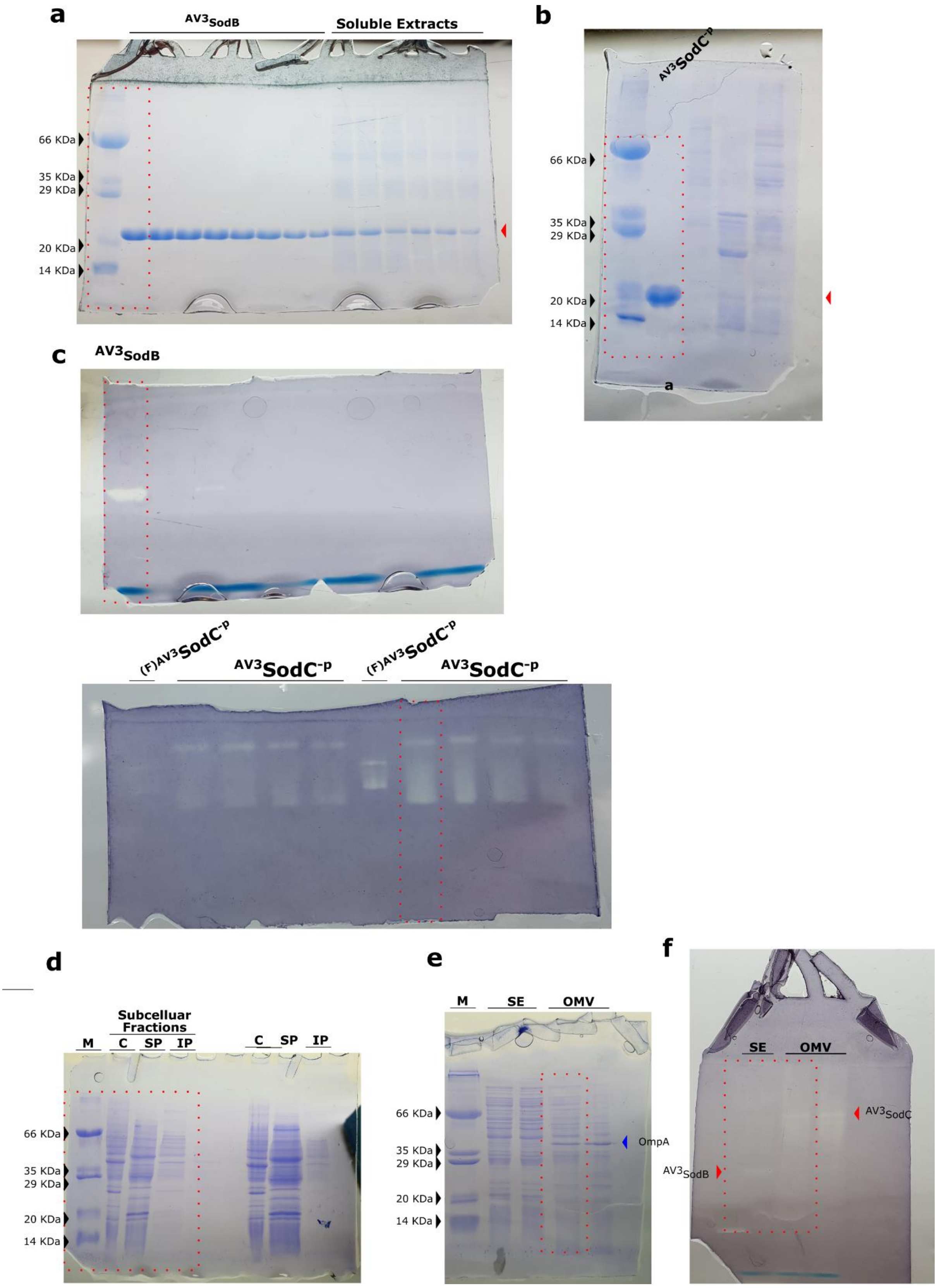
a
AV3SodB
Soluble Extracts
66 KDa
35 KDa
29 KDa
20 KDa
14 KDa
b
AV3SodC-P
66 KDa
35 KDa
29 KDa
20 KDa
14 KDa
a
c
AV3SodB
(F)AV3SodC-P
AV3SodC-P
(F)AV3SodC-P
AV3SodC-P
d
Subcelluar Fractions
M
C
SP
IP
C
SP
IP
66 KDa
35 KDa
29 KDa
20 KDa
14 KDa
e
M
SE
OMV
66 KDa
35 KDa
29 KDa
20 KDa
14 KDa
OmpA
f
SE
OMV
AV3SodC
AV3SodB

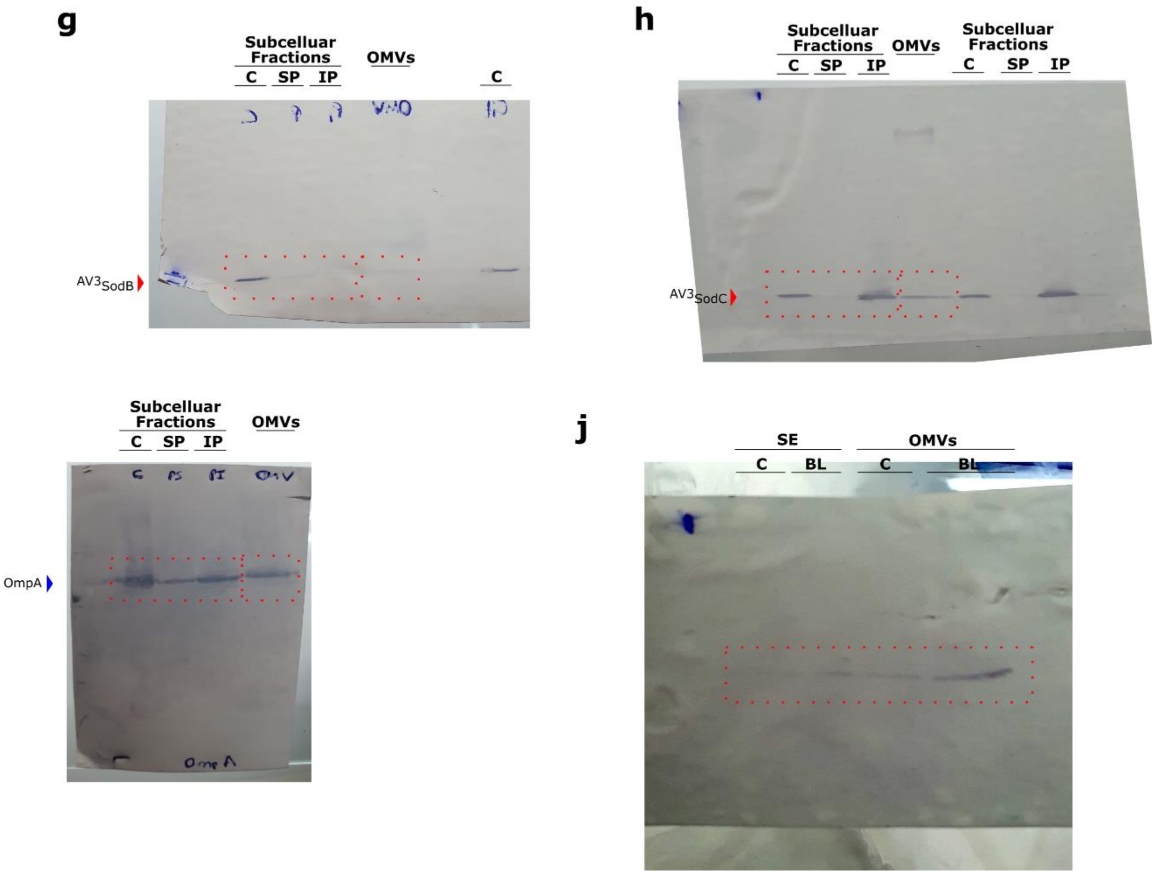


**Figure S5. Full-length gels/blots corresponding to the Figures 3 and 5 from the main text.** (a) SDS-PAGE for the purified $^{AV3}$SodB and (b) for $^{AV3}$SodC$^{-p}$. (c) nondenaturing polyacrylamide gels for $^{AV3}$SodB (up) and $^{AV3}$SodC$^{-p}$ (down). The SOD activity from the enzyme with the previous Thrombin treatment ((F)$^{AV3}$SodC$^{-p}$) is also present. (d) SDS-PAGE from the cytosolic (C), soluble and insoluble periplasmic (SP and IP, respectively) fractions and (e) soluble extracts (SE) and OMVs from *Acinetobacter sp.* Ver3. (f) Nondenaturing polyacrylamide gels for the SE and OMV. (g) and (h), Western Blot assay of cytosolic (C), soluble and insoluble periplasmic (SP and IP, respectively) fractions (7 µg of total proteins) (duplicate in the case of $^{AV3}$SodC$^{-p}$), and OMVs (15 µL from a 500X culture concentrate) obtained from *Acinetobacter sp.* Ver3. Specific antibodies raised against $^{AV3}$SodB, $^{AV3}$SodC (red arrows) and (i) OmpA (blue arrow) were used. (j) Anti-$^{AV3}$SodC immunoblot of a soluble extract (SE, 7 µg of total proteins) and OMVs (15 µL from a 500X culture concentrate) of *Acinetobacter sp*. Ver3 grown over 26 h in the presence of blue light (BL, 5 µmol.m$^{-2}$.s$^{-1}$). A control (C) culture was also included. The red boxes correspond to the cropped images displayed figures of the main text.
